# The prevalence of sedentary behavior among university students in Saudi Arabia

**DOI:** 10.1186/s12889-024-18107-7

**Published:** 2024-02-26

**Authors:** Mohammad A. Alahmadi, Khalid H. Almasoud, Amani H. Aljahani, Naweed S. Alzaman, Omar M. Al-Nozha, Osama M. Alahmadi, Rola A. Jalloun, Eman M. Alfadhli, Jomana M. Alahmadi, Areeg A. Zuair, Naif S. Alzahrani, Ahmed A. Alahmdi, Mansour A. Alghamdi, Abdulaziz A. Aldayel, Sulaiman O. Aljaloud, Obead M. Alharbi, Anwar Al-Nuaim, Shokrya S. Alshqaq, Basim S. Alsaedi, Afaf Alrashidi, Osama A. Alamri, Abdulwahed S. Alshaikhi, Fahad J. Al-Thumali, Khaled A. Alshdokhi, Abdulmohsen Bin Awn, Ali Abdullah Jifri, Osama Aljuhani, Khalid S. Aljaloud, Munirah Fayez Al-Mudarra, Mohammed G. A. Ansari, Nasser M. Al-Daghri

**Affiliations:** 1 Sport Science and Physical Activity Department, Taibah University, Madinah, Saudi Arabia; 2 Physical Sport Science Department, Princess Nourah bint Abdulrahman University, Riyadh, Saudi Arabia; 3 Medicine Department, College of Medicine, Taibah University, Madinah, Saudi Arabia; 4 College of Medicine, Dar Al Uloom University, Riyadh, Saudi Arabia; 5 Nutrition and Food Science Department, Taibah University, Madinah, Saudi Arabia; 6 College of Pharmacy, King Saud University, Riyadh, Saudi Arabia; 7 Community Health Nursing Department, Taibah University, Madinah, Saudi Arabia; 8 Medical Surgical Nursing Department, Taibah University, Madinah, Saudi Arabia; 9 College of Medicine, Al-Rayan Colleges, Madinah, Saudi Arabia; 10 Anatomy Department, King Khalid University, Abha, Saudi Arabia; 11 Exercise Physiology Department, King Saud University, Riyadh, Saudi Arabia; 12 Department of Curriculum and Instruction, Collage of Education, Qassim University, Burayday, Saudi Arabia; 13 Physical Education Department, Education College, King Faisal University, Hofuf, Saudi Arabia; 14 Department of Mathematics, Jazan University, Jazan, Saudi Arabia; 15 Department of Statistics, College of Science, University of Tabuk, Tabuk, Saudi Arabia; 16 Department of Mathematics, College of Science & Arts, King Abdulaziz University, Jeddah, Saudi Arabia; 17 Department of Sport Sciences, Taif University, Taif, Saudi Arabia; 18 Department of Sport Sciences and Physical Activity, College of Education, University of Hail, Hail, Saudi Arabia; 19 Department of General Curricula and Instruction, College of Education, King Saud University, Riyadh, Saudi Arabia; 20 Department of Sport Science, College of Sport Sciences, University of Jeddah, Jeddah, Saudi Arabia; 21 Department of Physical Education, College of Sports Science and Physical Activity, King Saud University, Riyadh, Saudi Arabia; 22 Department of Home Economics, College of Education, Prince Sattam bin Abdulaziz University, Wadi Al-Dawasir, Saudi Arabia; 23 Chair for Biomarkers of Chronic Diseases, Biochemistry Department, King Saud University, Riyadh, Saudi Arabia

**Keywords:** Physical activity, Sedentary behavior, Sedentary lifestyle, Questionnaire based study, Survey study

## Abstract

**Background:**

A considerable body of research has demonstrated that reducing sitting time benefits health. Therefore, the current study aimed to explore the prevalence of sedentary behavior (SB) and its patterns.

**Methods:**

A total of 6975 university students (49.1% female) were chosen randomly to participate in a face-to-face interview. The original English version of the sedentary behavior questionnaire (SBQ) was previously translated into Arabic. Then, the validated Arabic version of the SBQ was used to assess SB. The Arabic SBQ included 9 types of SB (watching television, playing computer/video games, sitting while listening to music, sitting and talking on the phone, doing paperwork or office work, sitting and reading, playing a musical instrument, doing arts and crafts, and sitting and driving/riding in a car, bus or train) on weekdays and weekends.

**Results:**

SBQ indicated that the total time of SB was considerably high (478.75 ± 256.60 and 535.86 ± 316.53 (min/day) during weekdays and weekends, respectively). On average, participants spent the most time during the day doing office/paperwork (item number 4) during weekdays (112.47 ± 111.11 min/day) and weekends (122.05 ± 113.49 min/day), followed by sitting time in transportation (item number 9) during weekdays (78.95 ± 83.25 min/day) and weekends (92.84 ± 100.19 min/day). The average total sitting time of the SBQ was 495.09 ± 247.38 (min/day) and 58.4% of the participants reported a high amount of sitting time (≥ 7 hours/day). Independent t-test showed significant differences (*P* ≤ 0.05) between males and females in all types of SB except with doing office/paperwork (item number 4). The results also showed that male students have a longer daily sitting time (521.73 ± 236.53 min/day) than females (467.38 ± 255.28 min/day). Finally, 64.1% of the males reported a high amount of sitting time (≥ 7 hours/day) compared to females (52.3%).

**Conclusion:**

In conclusion, the total mean length of SB in minutes per day for male and female university students was considerably high. About 58% of the population appeared to spend ≥7 h/day sedentary. Male university students are likelier to sit longer than female students. Our findings also indicated that SB and physical activity interventions are needed to raise awareness of the importance of adopting an active lifestyle and reducing sitting time.

## Background

According to the most recent estimates reported by the World Health Organization (WHO), 1.4 billion individuals (or 27.5% of adults) do not engage in the required physical activity [1]. However, physical inactivity differs from being sedentary. Sedentary behavior (SB) is defined as any sedentary doings during waking time, such as sitting or leaning, with minimum energy expenditure (≤1.5 metabolic equivalents (MET)) [2]. SB is different from physical inactivity which is defined as insufficient levels of physical activity or not meeting recommended levels of daily physical activity of moderate to high intensity [3, 4]. SB has become a concern in recent decades, especially among adults [5]. If the current behaviors are not reversed during youth, the burden of disease and injuries will rise as they approach adulthood [6]. Research has shown that sedentary lifestyles increase the risk of chronic illnesses, regardless of body weight [7–9] and physical activity levels [10, 11]. In Saudi adolescents, high amounts of sitting time (more than 2 hours) have been found among male students (84%) and female students (91.2%) [12]. It was also found that the majority of 456 female participants (> 85%) spent more time in sedentary activity (> 3 h/day) [13].

In Saudi Arabia, the research area of SB is relatively new in the medical field and is yet to be fully explored [14]. SB has been added to the latest WHO 2020 global guidelines on physical activity and SB [15]. More recently, the American Diabetes Association has released the Standards of Care in Diabetes—2024 which stated that all individuals are recommended to decrease the amount of time spent in daily SB and interrupt prolonged sitting every 30 minutes [16]. Thus, more research with precise measurements is needed to understand the complete picture of SB and its patterns. In Saudi Arabia, the most common tool to measure SB is a questionnaire, which consists of a single domain (i.e., screen time), including watching TV, using the internet, and playing electronic games [14, 17–22]. A recent meta-analysis showed that SB is more likely to be underestimated when SB questionnaires with few items were used, compared to multi-domain SB questionnaires such as the Sedentary Behavior Questionnaire (SBQ) [23]. The SBQ has been widely used [24], and the Arabic version of the SBQ became available with acceptable levels of validity and reliability to assess SB among Saudi males and females aged between 18 and 30 [25]. Test and retest reliability for all the Arabic SBQ items ranged from 0.64 to 0.87 [22]. The prevalence of SB using the Arabic SBQ in university students in Saudi Arabia has not been determined. Therefore, the current study aimed to assess the prevalence of SB and its patterns using the Arabic version of the SBQ among male and female university students across Saudi Universities.

## Methods

### Study design and population

**Fig. 1:**
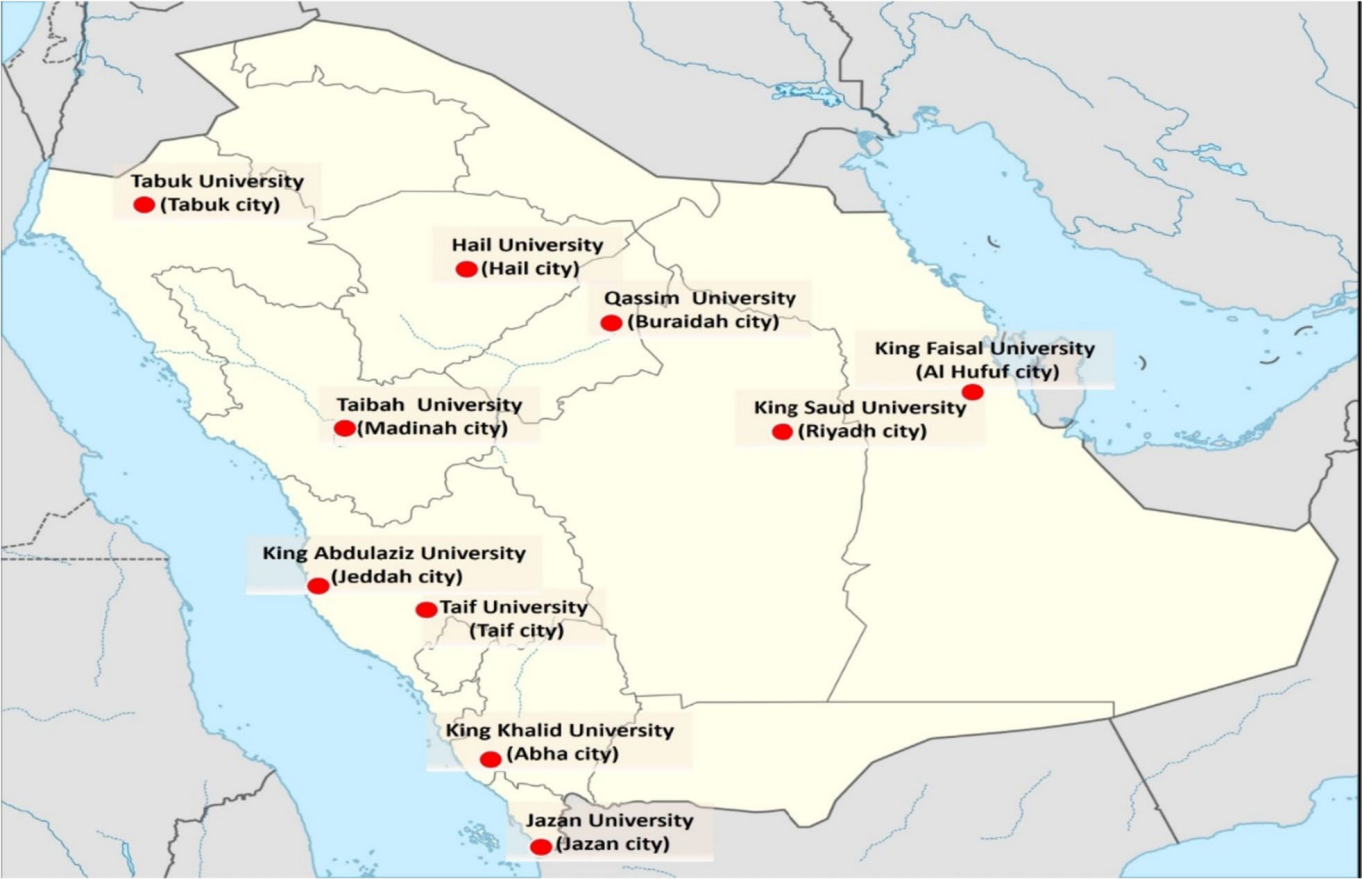
Map of universities and cities involved in the prevalence study in Saudi Arabia

The present study was countrywide, with a sample size of 7393 male and female university students. This study is an adult (aged between 18 and 35 years old), multi-city study, including 10 city-regions in Saudi Arabia. The present analyses included 10 university sites that collected SB data using a validated questionnaire. University students were randomly selected and recruited from different colleges such as Health, Sciences, Art and Humanities. Inclusion criteria included university students age ranged from > 18 and to 35. Participants were excluded from the study if they were not enrolled at the university. Students with physical or mental handicap were excluded. Students with learning difficulties were also excluded. The inclusion and exclusion criteria were indicated on the recruitment paper. The students were invited to partake in the study by completing the Arabic version of the SBQ via a face-to-face interview. The present study was conducted in 10 universities in 10 different cities of Saudi Arabia (Fig. 1) from spring 2022 to spring 2023. The location of each university and city is displayed in Fig. 1. The study protocol and procedures conformed to international ethical guidelines. Ethical approval of this study was obtained from The Ethics Committee of Taibah University Review Committee (TUCDREC/02033021/MAlahmadi). Participants were fully informed about the purpose and procedures of the study before reading and signing the informed consent form.

The sample size was chosen based on guidelines that suggest that a precision of 5% is used if the prevalence of SB is going to be between 10 and 90% [26]. Therefore, when the prevalence of SB is assumed to be 0.6 with a precision of 0.05, the adequate sample size will be 368 participants per university. An additional 15% was allowed as a non-response rate. The final sample size is 423 participants per university.

To further explain, the formula used for calculating the adequate sample size in the current study was the following [26]:

$$\textrm{n}={\textrm{Z}}^2\textrm{P}\left(1-\textrm{P}\right)/{\textrm{d}}^2$$

Where n is the sample size, Z is the statistic corresponding to the level of confidence (z = 1.96 at 95% CI), P is expected prevalence (expected prevalence (*P* = 0.60), and d is precision if 5%, d = 0.05). Therefore, if the sample size was computed, this yields 368 samples. Adding the 15% non-response rate resulted in a final sample size of 423 students per university.

## Data collection

Anthropometric data were also collected by asking participants about their height and weight to calculate body mass index (BMI). BMI is the ratio between weight in kilograms to height in meters squared.

## SB assessment

The SBQ was adopted [24] to assess the time spent in sedentary activities. SBQ has been translated into Arabic according to guidelines for the cross-cultural adaption process recommended by Beaton et al. (2000). The Arabic version of the SBQ became available with acceptable levels of validity and reliability to assess SB among Saudi males and females aged between 18 to 30 years old [25]. This previous study showed moderate to good reliability between test and retest for most of the Arabic version SBQ items and total score during weekdays (0.72 to 0.8) and weekends (0.64 to 0.87) [25]. The Arabic SBQ includes nine behaviors (watching television, playing computer/video games, sitting while listening to music, sitting and talking on the phone, doing paperwork or office work, sitting and reading, playing a musical instrument, doing arts and crafts, and sitting and driving/riding in a car, bus or train) on weekdays and weekends. The average total sitting time was calculated based on 7 days during weekdays and weekends. The average total sitting time of the SBQ (min/day) was calculated as follows:

$$\left[\left(\textrm{SB}\ \textrm{on}\ \textrm{weekdays}\times 5\right)+\left(\textrm{SB}\ \textrm{on}\ \textrm{weekend}\ \textrm{days}\times 2\right)\right]\div 7$$

The sitting time cut-off point for mortality risk was suggested to be approximately 7 hours/day [27]. Therefore, participants who reported ≥7 hours/day (≥ 420 minutes/day) were categorized as having higher sitting time [27]. Participants who reported more than 18 hours (approximately the maximum waking hours per day when sleeping duration is 6 hours) of total SB per day were considered implausible values and outliers. Therefore, participants who reported more than 18 hours of total SB per day were excluded. From a total sample of 7393, 6% of participants (i.e. 418 participants) provided implausible responses to the Arabic SBQ and were excluded from the final dataset.

## Data analysis

The Statistical Package for Social Sciences (SPSS Ins., Chicago, IL, USA) version 25 was used for data analysis. Descriptive statistics were presented as mean values and standard deviation (SD) for the Arabic SBQ items, total sitting time during weekdays and weekends, and the average sitting time among university students. The results of the prevalence of SB among university students were presented as percentage values. Independent t-test was utilized to analyze the differences in sitting time between genders. Multiple comparison method for means was used to determine differences between universities in the average sitting time. A post hoc LSD analysis was also used to examine pairwise comparisons. The significance level was set at ≤0.05.

## Results

A total of 6975 university students (49.1% female) participated in this study. Table 1 shows the mean and SD of the characteristics of University student participants. The mean value of BMI of the students population was within normal range (23.06 ± 4.85 kg/m^2^). With the exception of age, anthropometric characteristics did differ between male and female students.

**Table 1:** Characteristics of participants (*N* = 6975)

Variables	Mean ± SD		
	Male (*n* = 3553)	Female (*n* = 3422)	Total Sample (*N* = 6975)
Age (year)	21.03 ± 1.99	21.08 ± 1.99	21.05 ± 1.99
Height (cm)	172.33 ± 6.55	158.14 ± 6.67	165.37 ± 9.70
Weight (kg)	71.22 ± 15.54	55.28 ± 1.59	63.43 ± 15.89
BMI (kg/m^2^)	23.97 ± 5.07	22.10 ± 4.42	23.06 ± 4.85

Table 2 represents the characteristics of participants in each university, total sitting time, average sitting time, and sitting time ≥ 7 h/day (%) in 10 universities. Table 2 also shows the prevalence of SB among university students, ranging from 38.3 to 76.7%. High sitting time was defined as more than 7 hours (420 minutes) of sitting per day accumulated by students.

**Table 2:** Characteristics of participants, total sitting time, average sitting time, and percentage of students’ sitting time ≥ 7 h/day (%) in 10 universities

Universities	Variables (Mean ± SD)							Sitting time ≥ 7 h/day (%)
	Age (year)	Height (cm)	Weight (kg)	BMI (kg/m^2^)	Total sitting time of SBQ (min/day)		Average total sitting time of SBQ (min/day)^a^	
					Weekdays	Weekends		
1- Taibah University (Madinah) (*n* = 839, M = 373, F = 466)	20.42 ± 1.73	163.50 ± 9.55	60.69 ± 15.87	22.53 ± 4.69	483.75 ± 236.12	491.53 ± 261.05	485.97 ± 226.84	56.7%
2- Qassim University (*n* = 610, M = 285, F = 325)	20.60 ± 2.00	164.16 ± 9.44	62.08 ± 17.51	22.87 ± 5.45	411.09 ± 246.76	453.41 ± 327.84	423.18 ± 247.70	47.5%
3- University of Hail (*n* = 1034, M = 530, F = 504)	21.23 ± 1.24	166.96 ± 8.83	63.67 ± 13.41	22.75 ± 4.13	439.07 ± 182.49	507.39 ± 217.67	458.59 ± 174.72	55.7%
4- Jazan University (*n* = 569, M = 279, F = 290)	21.46 ± 2.13	162.38 ± 9.55	60.71 ± 14.42	22.87 ± 4.33	470.73 ± 284.06	519.15 ± 320.51	484.56 ± 278.44	54.1%
5- King Abdulaziz University (*n* = 538, M = 270, F = 268)	20.78 ± 2.05	165.96 ± 9.35	62.35 ± 14.97	22.50 ± 4.45	493.71 ± 283.25	631.39 ± 433.41	533.05 ± 275.56	64%
6- King Faisal University (*n* = 673, M = 355, F = 318)	21.12 ± 1.88	164.83 ± 9.65	65.41 ± 20.18	23.93 ± 6.76	617.49 ± 250.52	581.21 ± 276.47	607.12 ± 234.32	76.7%
7- King Saud University (*n* = 781, M = 395, F = 386)	21.33 ± 3.20	167.06 ± 9.21	67.86 ± 15.64	24.21 ± 4.85	454.81 ± 252.03	480.19 ± 302.02	462.06 ± 235.09	50.4%
8- Tabuk University (*n* = 786, M = 497, F = 289)	21.24 ± 1.77	167.09 ± 9.09	67.21 ± 17.97	23.90 ± 5.35	544.04 ± 251.52	644.87 ± 289.99	572.85 ± 241.84	71.5%
9- Taif University (*n* = 557, M = 278, F = 279)	20.95 ± 1.89	167.80 ± 10.04	58.28 ± 12.89	20.56 ± 3.33	546.67 ± 270.46	618.47 ± 384.70	567.19 ± 255.75	67.1%
10- King Khalid University (*n* = 588, M = 291, F = 297)	21.42 ± 1.89	162.66 ± 10.32	64.53 ± 12.29	24.30 ± 3.43	327.06 ± 235.61	461.54 ± 333.56	365.48 ± 245.65	38.3%

^a^The average total sitting time of SBQ (min/day) = [(SB on weekdays × 5) + (SB on weekend days × 2)]/7

As can be seen in Table 2, students from King Faisal University reported higher time of SB in total (mean ± SD, 607.12 ± 234.32) and during weekdays (mean ± SD, 617.49 ± 250.52). In contrast, students from King Khalid University reported the lowest time of SB in total (mean ± SD, 365.48 ± 245.65) and during weekdays (mean ± SD, 327.06 ± 235.61). Students in Tabuk University reported a higher time of SB during weekends (mean ± SD, 644.87 ± 289.99 min). The results also showed a significant difference in sitting time between different universities, and post hoc analysis revealed significant differences between most universities. The most important finding was that the prevalence of SB among university students ranged from 38.3% in King Khalid University to 76.7% in King Faisal University (the mean difference was 241.6, 95%CI 215.3, 267.9], *P* = 0.000). Only a few universities exhibited no differences. For example, the average total sitting time did not differ between Taibah university and Jazan University (*P* = 0.913). No difference was also found between King Saud University with both Hail University (*P* = 0.758) and Jazan University (*P* = 0.086). Tabuk University with Taif University also did not differ in sitting time (*P* = 0.668).

Finally, it is important to note that no correlations were found between BMI and total sitting time during weekdays (*P* = 0.868) and weekends (*P* = 0.156), and the average total sitting time of the SBQ (*P* = 0.520).

Table 3 presents the Arabic SBQ items and total scores of SB across participants. Overall, the mean total SB (min/day) was 478.75 ± 256.60 and 535.86 ± 316.53 during weekdays and weekends, respectively. The total mean of doing office/paperwork (item number 4) accounted for the highest amount of time spent (min/day) during weekdays (112.47 ± 111.11) and weekends (122.05 ± 113.49). Sitting time in transportation (item number 9) was the second highest type of SB during weekdays and weekends, accounting for 78.95 ± 83.25 and 92.84 ± 100.19 min/day, respectively. The average mean time spent engaged in these behaviors was higher during weekends (535.86 ± 316.53 min/day) than on weekdays (478.75 ± 256.60 min/day). Finally, the average total sitting time of the SBQ was 495.09 ± 247.38 (min/day) and 58.4% of the participants reported a high amount of sitting time (≥ 7 hours/day). Significant differences (*P* ≤ 0.05) were found between males and females in all types of SB except with doing office/paperwork (item number 4). Males also tended to sit more than females as the average total sitting time was higher in males (521.73 ± 236.53 min/day) compared to females (467.38 ± 255.28 min/day), *P* = 0.000. Table 3 also shows that 64.1% of the males reported a higher amount of sitting time (≥ 7 hours/day) compared to females (52.3%).

**Table 3:** The mean ± SD of the Arabic SBQ items, total sitting time, average total sitting time of SBQ, and sitting time ≥ 7 h/day (%) (*N* = 6975)

SBQ Items	Male		Female		Total Sample	
	Weekdays	Weekends	Weekdays	Weekends	Weekdays	Weekends
1.TV (min/day)	57.80 ± 71.87^**^	77.79 ± 85.13	61.90 ± 80.26	72.06 ± 89.77	59.82 ± 76.12	74.98 ± 87.48
2. Computer/games (min/day)	79.84 ± 87.43^**^	107.95 ± 107.01	40.44 ± 68.94	49.66 ± 79.40	60.50 ± 81.31	79.39 ± 9 8.88
3.Sit and listen to music (min/day)	40.53 ± 61.59^**^	47.74 ± 65.91	47.97 ± 68.48	51.79 ± 71.38	44.18 ± 65.16	49.72 ± 68.67
4.Office/paperwork (min/day)	110.83 ± 109.19	121.02 ± 111.93	114.18 ± 113.06	123.12 ± 115.10	112.47 ± 111.11	122.05 ± 113.49
5.Reading (min/day)	46.63 ± 67.49^**^	35.86 ± 59.83	58.76 ± 81.04	46.08 ± 69.89	52.58 ± 74.68	40.87 ± 65.15
6. Sit talk on the phone (min/day)	33.65 ± 53.56^**^	30.33 ± 51.60	48.03 ± 70.91	46.22 ± 70.07	40.71 ± 63.08	38.11 ± 61.85
7. Play musical instrument (min/day)	9.38 ± 30.81^**^	14.59 ± 41.55	14.30 ± 43.43	16.01 ± 49.80	11.80 ± 37.62	15.29 ± 45.79
8. Arts and crafts (min/day)	18.45 ± 44.97^**^	23.55 ± 53.35	25.12 ± 52.34	28.53 ± 59.06	21.73 ± 48.85	26.00 ± 56.28
9. Sitting driving/riding in a car, bus, or train (min/day)	101.76 ± 84.87^**^	125.54 ± 101.43	49.13 ± 72.37	58.81 ± 86.65	78.95 ± 83.25	92.84 ± 100.19
Total sitting time of SBQ (min/day)	497.81 ± 245.65^**^	581.55 ± 299.00	458.97 ± 266.09	488.42 ± 327.12	478.75 ± 256.60	535.86 ± 316.53
Average total sitting time of SBQ (min/day) *	521.73 ± 236.53^**^		467.38 ± 255.28		495.09 ± 247.38	
Sitting time ≥ 7 hours/day (%)	64.1%		52.3%		58.4%	

*The average total sitting time of SBQ (min/day) = [(SB on weekdays × 5) + (SB on weekend days × 2)]/7

**P ≤ 0.05 between males and females

Table 4 shows the mean difference between males and females in which male students tended to sit more during weekdays than female students (mean difference = 38.83, 95%CI 26.80, 50.86, *P* = 0.001). During weekends, however, female students tended to sit more than male students (mean difference = 93.12, 95%CI 78.40, 107.85, *P* = 0.001). In total, however, male students spent more time in sitting than female students (mean difference = 54.34, 95%CI 42.80, 65.89), *P* = 0.001).

**Table 4:** Mean Difference between males and females in Arabic SBQ items, and total sitting time during weekdays and weekends, and average total sitting time

SBQ Items	Mean Difference, 95% CI**	
	Weekdays	Weekends
1.TV (min/day)	−4.10[− 7.68, − 0.51]	5.73[1.62, 9.85]
2. Computer/games (min/day)	39.39[35.70, 43.0]	58.28[53.85, 62.70]
3.Sit and listen to music (min/day)	−7.43[− 10.50, − 4.36]	− 4.04[− 7.28, − 0.80]
4.Office/paperwork (min/day)	−3.35[− 8.57, 1.86]	−2.10[− 7.45, 3.24]
5.Reading (min/day)	−12.12[− 15.64, − 8.61]	−10.21[− 13.28, − 7.14]
6. Sit talk on the phone (min/day)	−14.37[− 17.33, 11.40]	−15.88[− 18.79, − 12.97]
7. Play musical instrument (min/day)	−4.91[− 6.69, − 3.14]	−1.41[− 3.58, 0.70]
8. Arts and crafts (min/day)	−6.67[− 8.97, − 4.37]	−4.97[− 7.63, − 2.31]
9. Sitting driving/riding in a car, bus, or train (min/day)	52.65[48.93, 56.33]	66.72[62.28, 71.15]
Total sitting time of SBQ (min/day)	38.83[26.80, 50.86]	93.12[78.40, 107.85]
Average total sitting time of SBQ (min/day) *	54.34[42.80, 65.89]	

*The average total sitting time of SBQ (min/day) = [(SB on weekdays × 5) + (SB on weekend days × 2)]/7

**CI Confidence interval

## Discussion

The current study has described the prevalence of SB and its patterns among male and female university students. In general, the prevalence of SB in Saudi Arabia is considerably high, ranging from 47 to 98%. The range of the prevalence of SB depends on factors such as gender and the cut-off point used for classifying SB. In a recent study, the prevalence of SB among Saudi females was 7.5 ± 3.6 h/day and about 98% of them spent more than 2 hours per day engaged in SB [21]. According to The American Academy of Pediatrics (AAP), the recommended amount of sedentary time per day is no more than 2 hours. Our study found that 94.8% of university men and women spend more than 2 hours daily in sedentary activity. It is known that excessive sitting time almost doubles the risk of type 2 diabetes [28]. Among studies conducted in Saudi Arabia, 2 hours per day of SB was most commonly cut off, according to the AAP [12, 18, 29, 30]. Only one study has used the cut-off point of 7 hours per day among Saudi females [21]. It was found that 47% of Saudi females spent more than 7 hours per day in sedentary activities [21]. This finding was similar to ours; more than half (58.4%) of our university students spent more than 7 hours daily in SB. Unfortunately, this may increase their risk of adverse health outcomes, such as cardiovascular disease, type 2 diabetes, cancer, and mortality [31, 32].

Our study also found that the average daily sedentary time was 8.3 hours among male and female university students. Similar results of average sedentary time among adults (8.65 h per day) were found in a study that included 10 countries [33]. In this study, SB was measured directly by an objective method (i.e., accelerometer), which was worn by the participants (5712 adults aged 18–65 years old) for 7 days [30].

A recent survey of university students found that students spend far too much time sitting down, often between 5 to 8 hours each day [34]. Our data showed that a large proportion (58.4%) of Saudi university students appear to have SB time of ≥7 h/day (420 min/day). When a cut-off value of ≥8 h/day (480 min/day) was used, the present study still found a high level of SB (49.4%) among university students in Saudi Arabia. In a study with a cut-off similar to our study, it was found that 47.7% of the population in Singapore had SB of ≥7 h/day [35]. However, the prevalence of SB was higher (about 60%) among Singaporeans aged 18–34 [35].

The present study also found a significant difference in sitting time across 10 universities. The prevalence of SB among university students (≥7 h/day) ranged from 38.3% in King Khalid University, Southern Saudi Arabia, to 76.7% at King Faisal University, Eastern Saudi Arabia. The mean difference was 241.6 min/day. This means that students at King Faisal University spent approximately 4 more hours per day sitting than those at King Khalid University. The Eastern region of Saudi Arabia is a wealthy part where the main oil companies are located, and of course, the current modernization may lead to an increase in the predominance of sedentary lifestyles in the Saudi adult population [36]. A cross-sectional study was conducted in Saudi adults (aged 38 ± 8 years) in Eastern Saudi Arabia found significant correlations between certain lifestyle behaviours and health risk factors [37]. Interestingly, adults who lived in Eastern Saudi Arabia and earned more than 10,000 SAR a month were 1.2 times more likely to have metabolic syndrome [37].

Based on prior studies and theory, BMI was assumed to be negatively correlated with time spent engaging in SB [38–40]. The relationship between sitting time with BMI in adults are contradictory. For example, while one study using SBQ found positive associations between BMI and total sitting time in overweight adults [21], another study found an inverse correlation between BMI and total sitting time of the Arabic SBQ in non-overweight adults [25]. The difference in body weight status may explain the contradictory results between the studies. When an object device was used to assess SB, no correlations were found between sedentary time, measured by accelerometers, and BMI [30]. The present study found no significant associations between SBQ items and BMI. The inconsistency between studies can be explained by the fact that BMI is affected by a number of factors other than sitting time, such as physical activity, energy intake, and heredity [41].

One of the most significant determinants of sitting time was thought to be gender. Numerous research revealed that the amount of sitting time differed by gender, with male individuals sitting more often than female ones [42]. Our current study found a significant difference between genders regarding the sitting time of male and female university students. The average total sitting time was greater in males (8.41 h/day) than in females (7.47 h/day), suggesting that male university students tend to sit more than female students. We also found that there were higher amounts of sitting time (more than 7 hours/day) among male university students (64.1%) compared to female university students (52.3%). It is evident that our students exceed the recommended sitting time per day. Adult mortality risk appears to rise with sitting periods of more than 7 or 8 hours per day [27]. Also, adults who spend more than 10 hours per day sitting down have a 29% higher risk of dying prematurely than those who spend less than 6 hours per day sitting down [43].

The most common tool to measure SB in Saudi Arabia is a questionnaire consisting of a single domain (i.e., screen time), including three items: watching TV, using the internet, and playing electronic games [14, 17–22]. However, the Arabic SBQ with nine items (during weekdays and weekend) conducted in males and females is more likely to give a bigger picture regarding SB and its types. An interesting issue regarding SB and time spent in various sedentary activities is the patterns that may differ by gender. Growing evidence suggests that gender differences in SB exist [44]. For example, it was found that males spent more time playing video/computer games, while girls spent more time in leisure reading [44].

In our study, male university students spent more time playing computer games during weekdays (79.84 ± 87.43 min/day) and weekends (107.95 ± 107.01 min/day) than female university students. Interestingly, we also noted that SB activities such as reading, talking on the phone, playing musical instruments, and doing arts and crafts were the most common type of SB among female university students compared to male university students. Male university students spent more time driving/ riding a car (item number 9) than females. This finding is similar to a study that found males were more involved in activities like motor transport than females [45]. Due to the high correlation between driving and leisure activities, male drivers tend to drive with friends for fun in the evening and at night [46]. It is also important to note that male students in the present study tended to be more sedentary during weekends than weekdays. The effect of weekdays and weekends on sedentary time was already established [47]. A recent study found that the time spent using a computer and the internet was higher during weekends than on weekdays [48]. Our results are consistent with such findings. The male university students spent more time playing computer/games during weekends (107.95 ± 107.01 min/day) than on weekdays (79.84 ± 87.43 min/day).

Finally, our study has strengths. First, it is one of the most extensive studies using face-to-face interview questionnaires conducted in 10 different cities in Saudi Arabia to explore SB among students with more than 7000 male and female university students. Second, our study is the first in the Arab countries using a validated multi-domain SB questionnaire (i.e., the Arabic SBQ), compared to a questionnaire with few items. However, the current study has some limitations. While the SBQ approach has achieved universal acceptance as a tool for assessing sedentary lifestyles and has been extensively utilized in adult studies, several limitations must be acknowledged. First, self-reporting was the only data collection method, which may cause bias and affect the results. Second, the present study was conducted only on male and female university students aged between 18 to 30 years old. Consequently, it’s possible that the sample from this study doesn’t accurately reflect the population and cannot be representative. Therefore, further research is needed to validate our results and extend the present findings.

## Conclusion

The total mean length of SB for Saudi university students was high, with approximately 58% of students spending 7 hours per day sedentary. Male university students tend to have longer sitting times than females. Our findings indicated the need to increase awareness of the importance of adapting an active lifestyle. Our university students should be encouraged to decrease their involvement in SB to promote a healthy lifestyle and to facilitate the secondary prevention of chronic diseases by noting strategies and coordinating efforts at all levels (family, university, community, and government).

## Data Availability

Data will be available upon request from the corresponding author.

## Abbreviations

AAP: American academy of pediatrics
BMI: Body mass index
CI: Confidence interval
MET: Metabolic equivalents
SB: Sedentary behavior
SBQ: Sedentary behavior questionnaire
SD: Standard deviation
WHO: World health organization
